# The PRMT5-LSD1 axis confers Slug dual transcriptional activities and promotes breast cancer progression

**DOI:** 10.1186/s13046-022-02400-7

**Published:** 2022-06-02

**Authors:** Jianchao Zhang, Xiaokai Fan, Yunfan Zhou, Liang Chen, Hai Rao

**Affiliations:** 1 Department of Biochemistry, School of Medicine, Southern University of Science and Technology, Shenzhen, China; 2 Shenzhen Laboratory of Tumor Cell Biology, Center for Protein and Cell-Based Drugs, Institute of Biomedicine and Biotechnology, Shenzhen Institute of Advanced Technology, Chinese Academy of Sciences, Shenzhen, China

**Keywords:** Slug, PRMT5, LSD1, EMT, Methylation, Demethylation, Metastasis and breast cancer

## Abstract

**Background:**

Downregulation of epithelial markers and upregulation of mesenchymal markers are the characteristics of the epithelial to mesenchymal transition (EMT) program, which provides the metastatic advantage of breast cancer. However, the mechanism underlying the switch of EMT markers remains poorly understood.

**Methods:**

In this study, we used the affinity purification and mass spectrometry coupled approach to identify the interactome of Slug. CoIP, GST-pulldown, ChIP, Re-ChIP, qPCR and Immunoblot were used to investigate the underlying mechanism of Slug-PRMT5-LSD1 complex. The role of PRMT5 and LSD1 in breast cancer progression was evaluated both in vivo and in vitro.

**Results:**

Here we found that the transcription factor Slug associates with PRMT5 and LSD1 in a complex and facilitates the breast cancer invasion in vitro. Mechanistically, PRMT5 and LSD1 work with Slug to exert dual transcriptional activities to inhibit E-cadherin expression by PRMT5-catalyzed H4R3me2s and LSD1-mediated demethylation of H3K4me2 on the E-cadherin (CDH1) promoter, and activate vimentin (VIM) expression via PRMT5-driven H3R2me2s and LSD1-mediated removal of H3K9me2. Importantly, PRMT5 and LSD1 are coordinately expressed in breast cancer patients and pharmacologic perturbation of both PRMT5 and LSD1 shows a synergetic effect on the inhibition of breast tumor growth and metastasis in vivo.

**Conclusions:**

Our study suggests that PRMT5 and LSD1 function as a dual epigenetic modifier to promote Slug induced EMT program, suggesting that the inhibition of PRMT5 and LSD1 presents a potential therapeutic strategy against cancer metastasis.

**Supplementary Information:**

The online version contains supplementary material available at 10.1186/s13046-022-02400-7.

## Background

Accumulating evidences have demonstrated that the epithelial to mesenchymal transition (EMT) process plays a crucial role during malignant tumor metastasis, and is associated with generation of drug resistance and cancer stem cell. During the EMT, epithelial cells shed their epithelial characteristics, including loss of cell junctions and apical-basal polarity, reduction of epithelial markers, followed by the gain of mesenchymal properties including enhanced invasiveness, reorganization of cytoskeleton and increased expression of mesenchymal markers. EMT is tightly orchestrated by EMT-inducing transcription factors (e.g., Twist1, Twist2, Snail, Slug, ZEB1 and ZEB2) that alter gene expression to trigger the epithelial to mesenchymal phenotype conversion [1].

Slug (also known as SNAI2), a member of the SNAIL superfamily of zinc-finger transcription factors, plays a pivotal role in modulating the expression of genes responsible for the EMT during embryogenesis and cancer [2, 3]. Previous studies have linked Slug to the invasion, metastasis, drug resistance, tumor stemness, and poor prognosis in a variety of cancers [4–8]. Slug has been found to not only act as a transcriptional repressor to block the expression of epithelial markers (e.g., E-cadherin, occludin and Claudin-1) [9–11], but also directly activate the expression of ZEB1 [12], an inducer of EMT. Yet, how Slug works with other proteins to facilitate the EMT process through a dual regulation of transcriptional suppression and activation remains enigmatic. Better understanding of the regulatory mechanisms of Slug is essential to develop novel therapies to prevent tumor development and progression.

Other chromatin modifiers may be involved in assisting Slug. Protein arginine methyltransferase 5 (PRMT5) is a type II protein arginine methyltransferase, which is known to carry out symmetrical dimethylation on histone substrates (e.g., H4R3, H3R2, H3R8 and H2AR3) and non-histone proteins. PRMT5 is involved in gene silencing through the induction of repressive histone markers such as symmetrical dimethylation of H4R3 and H3R8, whereas PRMT5-catalyzed H3R2 symmetric dimethylation has been shown to mediate transcriptional activation [13]. PRMT5-mediated arginine methylation governs multiple biological processes including cell growth, apoptosis, stemness and motility [14]. PRMT5 is overexpressed in a wide variety of cancers, such as lung, breast, gastric and liver cancer [15]. PRMT5 has emerged as a possible cancer drug target and PRMT5 inhibitors are potent in clinical trials for blood and multiple solid malignancies.

It has been reported that Lysine-specific demethylase 1 (LSD1) associates with Snail and Slug, and is essential for Snail and Slug-mediated EMT and transcriptional inhibition of epithelial genes [16, 17]. LSD1 is the first histone demethylase discovered and specifically demethylates H3K4me1/2, H3K9me1/2 as well as some non-histone targets. LSD1 catalyzes demethylation at H3K4me1/2 resulting in gene silencing, whereas the removal of methyl groups from H3K9me1/2 by LSD1 is tied to transcriptional activation of target genes [18]. Elevated level of LSD1 has been found in diverse cancers [19]. Moreover, LSD1 is closely linked to many cellular processes including cell proliferation, survival and stemness [20–22]. Therefore, targeting LSD1 is becoming an attractive therapeutic option for the anti-cancer treatment. To date, numerous LSD1 inhibitors have been developed, some of which are currently being evaluated clinically for cancer therapy.

## Methods

### Reagents and plasmids

Antibodies used include: anti-Slug (9585, 1:1000), anti-LSD1 (2139, 1:2000), anti-myc (2276, 1:5000), anti-HA (3724, 1:10,000) from Cell Signaling Technology; anti-PRMT5 (ab109451, 1:2000), anti-H4R3me2s (ab5823, 1:1000), anti-H3K9me2 (ab1220, 1:1000) from Abcam; anti-GAPDH (60,004, 1:5000), anti-H3 (17,168, 1:2000), anti-H4 (16,047, 1:2000) from Proteintech; anti-E-cadherin (610,181, 1:2000), anti-vimentin (550,513, 1:10,000) from BD Biosciences; anti-Flag (ant-146-a, 1:5000) from PROSPEC; anti-H3R2me2s (A-3705, 1:1000) from Epigentek; anti-H3K4me2 (07–030, 1:2000) from Millipore; anti-Slug (sc-166476, 1:1000) from Santa cruz. Anti-Flag M2 affinity gel (A2220) and Flag peptide (F3290) were purchased from Sigma. SP2509 (HY-12635) and EPZ01566 (HY-12727) were obtained from MCE. The cDNA for wild-type or deletion mutants of Slug, PRMT5 and LSD1 was amplified by PCR and cloned into EcoRI site of a lentiviral vector CD532A with Flag, myc or HA tag, respectively or a bacterial expression vector pGEX-KG by homologous recombination using ClonExpress® II One Step Cloning Kit (Vazyme, China). The shRNA sequences targeting human Slug, PRMT5 and LSD1 were synthesized by RuiBiotech and subcloned into a pLKO.1 vector (Addgene). For knockdown experiments, two independent shRNA sequences were targeted for each gene and the one showing the higher silencing efficiency was used for the subsequent experiment. All DNA sequences were verified by sequencing. The shRNA targeting sequences used were: shSlug-1#, GAACTGGACACACATACAGTG; shSlug-2#, CAGACCCATTCTGATGTAAAG; shPRMT5-1#, GCCCAGTTTGAGATGCCTTAT; shPRMT5-2#, GCGTTTCAAGAGGGAGTTCAT; shLSD1-1#, CCACGAGTCAAACCTTTATTT; shLSD1-2#, GCTACATCTTACCTTAGTCAT.

### Cell culture

HEK293T, MCF10A and MDA-MB-231 cell lines were purchased from the American Type Culture Collection (ATCC, USA). HEK293T and MDA-MB-231 cells were maintained in DMEM (Thermo Scientific™) plus 10% fetal bovine serum (FBS) (ExCell Bio) and penicillin, streptomycin (Thermo Scientific™). MCF10A cells were cultured in DMEM/F12 supplemented with 5% horse serum (Gibco), 20 ng/mL EGF (R&D), 0.5 mg/mL hydrocortisone (Sangon Biotech, China), 100 ng/mL cholera toxin (Sigma), 10 mg/mL insulin (MCE) and penicillin/streptomycin. SUM159 cell line was purchased from Meisen Chinese Tissue Culture Collection (MeisenCTCC, China) and grown in RPMI1640 (Thermo Scientific™) supplemented with 10% FBS and penicillin, streptomycin. All the cell lines are routinely checked for morphological and growth characteristics. Mycoplasma testing of cell cultures was performed routinely using a MycoBlue Mycoplasma Detector Kit (Vazyme, China).

### Lentiviral production and infection

A lentiviral vector bearing the shRNA or cDNA of interest, along with a packing vector (psPAX2) and an envelope vector (pMD2.G) was co-transfected into HEK293T cells using polyethylenimine. Supernatants containing virus particles were collected at 48 h post transfection, and filtered through 0.45 μm filters to remove cell debris. The viruses were used to infect target cells grown in medium supplemented with 8 μg/mL polybrene. Infected cells were then obtained in the presence of 1 μg/mL puromycin.

### Immunoblot analysis

Cells were processed in lysis buffer (50 mM Tris–HCl, pH 8.0, 1% NP-40, 150 mM NaCl, 0.1% SDS, 0.5% sodium deoxycholate and 1 × complete protease inhibitor cocktail) on ice for 1 h and centrifuged at 16,000 g for 10 min at 4 °C to collect the supernatant. After the protein concentrations were measured by the BCA protein assay kit (Thermo Scientific™), samples with equal amounts of proteins were mixed with loading buffer and boiled. Protein lysates were resolved by SDS-PAGE and transferred to PVDF membranes. Then, the membranes were blocked in 5% non-fat milk for 1 h at room temperature, incubated with a primary antibody overnight at 4 °C, and then incubated with HRP-conjugated secondary antibodies for 1 h at room temperature. Subsequently, the membranes were probed with ECL reagent (Millipore) and proteins were visualized by a Tanon-5200 Automatic Chemiluminescence Imaging Analysis System (Tanon, China).

### Immunopurification and mass spectrometry

HEK293T cells expressing Flag-Slug were lysed in lysis buffer (50 mM Tris–HCl, pH 8.0, 0.2% NP-40, 150 mM NaCl, 2 mM EDTA and 1 × complete protease inhibitor cocktail) on ice for 30 min and centrifuged at 10,000 g for 10 min at 4 °C to collect the supernatant. Cell lysates extracted from about 5 × 10^8^ cells were incubated with 150 µL equilibrated anti-Flag M2-agarose beads for 1.5 h at 4 °C. After binding, the beads were washed with cold lysis buffer five times. Flag peptide (Sigma) was added into the resin to elute the Flag protein complex overnight at 4 °C and centrifuged at 8,000 g to collect the supernatant. Then the supernatant was resolved on 4–12% gradient gels (GenScript), stained using silver stain kit (Pierce), and subjected to LC–MS/MS (ThermoFisher Q Exactive mass spectrometer) sequencing.

### Co-immunoprecipitation

Cell lysates were obtained by incubating the cells in lysis buffer (50 mM Tris–HCl, pH 8.0, 0.2% NP-40, 150 mM NaCl, 2 mM EDTA and 1 × complete protease inhibitor cocktail) for 20 min at 4 °C, followed by centrifugation at 14,000 g for 15 min at 4 °C. Overall, 5% whole-cell extracts were used for input. The rest of the protein extracts were incubated with 2 μg control or specific antibodies overnight at 4 °C. Then, 10 μL of Protein G magnetic beads (Invitrogen) were added with further incubation at 4 °C for 2 h. Beads were then washed five times using the cold lysis buffer. The immunoprecipitates were boiled with 2 × SDS-PAGE loading buffer, separated on SDS-PAGE gels, followed by immunoblotting with various antibodies indicated.

### GST pull-down assay

GST fusion proteins were transformed in *E. coli* (BL21) and induced with 1 mM IPTG at 37 °C for 3 h. The cells were harvested by centrifugation at 4,000 rpm for 10 min. Then, the bacterial pellets were resuspended and lysed by sonication in cold PBS in the presence of complete protease inhibitor cocktail, followed by centrifugation to collect the supernatant at 12,000 g for 10 min at 4 °C. The cell lysates were applied to prepared glutathione-Sepharose 4B beads (GE Healthcare) and incubated for 2 h at 4 °C. Beads were washed five times using the cold PBS. Glutathione-Sepharose-bound GST fusion proteins were mixed with Flag-tagged fusion proteins, which were purified from HEK293T cells, and incubated for 2 h at 4 °C. The beads were then washed three times with wash buffer (50 mM Tris–HCl, pH 8.0, 0.2% NP-40, 150 mM NaCl, 2 mM EDTA). The bound proteins were eluted by boiling in 2 × SDS-PAGE loading buffer, followed by immunoblotting analysis. The purified GST fusion proteins were examined for the presence by coomassie brilliant blue staining.

### Chromatin immunoprecipitation (ChIP) and Re-ChIP

Cells were cross-linked with 1% formaldehyde for 10 min at 37 °C and stopped by 125 mM glycine at room temperature for 5 min. Then cells were washed with cold PBS, and resuspended in cell lysis buffer (1% SDS, 1 mM EDTA, 25 mM Tris–HCl, pH 8.0) for 30 min at 4 °C. Samples were centrifuged to obtain the supernatant. The supernatant was subjected to sonication to shear the chromatin between 100 and 500 bp. After centrifugation at 16,000 g for 10 min at 4 °C, the protein-DNA complexes were immunoprecipitated with 2 μg control or specific antibodies overnight at 4 °C, followed by further incubation with protein G magnetic beads for 2 h at 4 °C. Complexes were washed with low-, high-salt and LiCl wash buffer sequentially, followed by two washes with TE buffer at 4 °C. The complex was eluted by adding 100 μl elution buffer (1% SDS, 0.1 M NaHCO_3_) with rotation at 37 °C for 30 min twice. Then the reverse crosslinking was carried out by adding NaCl (0.2 M) and proteinase K (0.5 mg/ml) and incubated at 65 °C overnight. DNAs were purified using a DNA purification kit. The purified DNA was dissolved in ddH_2_O for the qPCR. For Re-ChIP, immune complexes were eluted from the first IP by incubation with 10 mM DTT at 37 °C for 30 min. Eluents were diluted 1:50 in dilution buffer (150 mM NaCl, 1% Triton X-100, 2 mM EDTA, 20 mM Tris–HCl pH 8.0), and subjected to Re-IP with the secondary antibodies. DNA template enrichment was analyzed by conventional PCR. The following primers were used for the E-cadherin promoter: 5’-GAACCCTCAGCCAATCAGC-3’ (forward) and 5’- CTGACTTCCGCAAGCTCACA-3’ (reverse); vimentin promoter: 5’- GAGGGGACCCTCTTTCCTAA-3’ (forward) and 5’-GAGAGTGGCAGAGGACTGGA -3’ (reverse); Claudin-1 promoter: 5’- AGCTCTGGTGCCTGGTCCT-3’ (forward) and 5’- GGTTTCAGGGCGGCTCACC-3’ (reverse); ZEB1 promoter: 5’-GCTGCGCGGGTCAGGTAGC-3’ (forward) and 5’-GTCTGGACTCCCCGGGGAGT-3’ (reverse).

### Quantitative real-time PCR (qPCR) and RT-PCR

Total RNA was isolated using FastPure Cell/Tissue Total RNA Isolation Kit V2 (Vazyme, China) and then reverse transcribed into cDNA using TransScript II One-Step gDNA Removal and cDNA Synthesis SuperMix (Transgen, China). RT-PCR was performed using 2 × Accurate Taq Master Mix (Accurate Biology, China). qPCR was performed using Taq Pro Universal SYBR qPCR Master Mix (Vazyme, China) on a QuantStudio 7 Flex Real-Time PCR system (ABI). GAPDH serves as an internal control in all experiments. The primer sequences used were as follows: E-cadherin: 5’- GACAACAAGCCCGAATT-3’ (forward) and 5’-GGAAACTCTCTCGGTCCA-3’ (reverse); vimentin: 5’- GAGAACTTTGCCGTTGAAGC-3’ (forward) and 5’-GCTTCCTGTAGGTGGCAATC-3’ (reverse); Slug: 5’-GGTCAAGAAGCATTTCAAC-3’ (forward) and 5’-GGTAATGTGTGGGTCCGA-3’ (reverse); PRMT5: 5’-GAGAATGCACCAACTACACA-3’ (forward) and 5’-ATTTCAAGAGCCACTGCAAT-3’ (reverse); LSD1: 5’-GAATTTGCTAATGCCACACC-3’ (forward) and 5’-GTATTCACAGCTATCACTTCAC-3’ (reverse); GAPDH: 5’-ATGACCCCTTCATTGACCTCA-3’ (forward) and 5’-GAGATGATGACCCTTTTGGCT-3’ (reverse); Claudin-1: 5’-TATTTCTTCTTGCAGGTCTGGCT-3’ (forward) and 5’-CTGGCATTGACTGGGGTCAT-3’ (reverse); ZEB1: 5’-TGCACTGAGTGTGGAAAAGC-3’ (forward) and 5’-TGGTGATGCTGAAAGAGACG-3’ (reverse).

### Immunohistochemical staining

Immunohistochemical staining was carried out to assay PRMT5 or LSD1 on the breast cancer tissue microarray (Avilabio, Xian, China). Specifically, the tissue sections were deparaffinized and rehydrated by heating the sample at 95 °C in Tris–EDTA buffer (pH 9.0) for 20 min. Endogenous peroxidase activity was blocked by peroxidase (ZSGB-BIO, China). The sections were blocked with goat serum and then mixed with anti-PRMT5 (Abcam, ab109451, 1:100) or anti-LSD1 antibody (CST, 2139, 1:50) at 4 °C overnight, followed by mixing secondary antibodies (Proteintech, China) for 1 h and developed with 3.3′-diaminobenzidine. Hematoxylin was used to counterstain the nuclei. Samples were scored by the H-score method that combines the intensity of staining and the percentage of positive cells. PRMT5 or LSD1 levels were scored on the following scale: Staining Intensity (SI): 0, no staining; 1, weak staining; 2, moderate staining; 3, intensive staining; Percentage of Positive Cells (PP): 0, no positive cells; 1, less than 10%; 2, 11–50%; 3, 51–80%; 4, over 80%. The immune reactive score (IRS): SI × PP (0–12). IRS < 4 was considered as negative or weak staining (Low), 6–8 as moderate staining (Middle) and 9–12 as strong staining (High). Scoring of the breast cancer samples was done in a blind manner by a board-certified pathologist.

### Transwell invasion assays

The cells starved with DMEM medium supplemented with serum free media overnight were seeded at a density of 5 × 10^4^ cells per well into Matrigel-coated invasion chambers (8-μm pore size, BD Biosciences). The lower chambers contained culture media containing 10% FBS. The wells were washed with PBS and fixed with 4% paraformaldehyde. The cells on the apical side of each insert were removed by scraping. Cells migrated to the basal side of the membrane were stained with 0.1% crystal violet and processed using Image-pro plus software.

### Mouse xenograft models

MDA-MB-231 cells that had been engineered to express firefly luciferase stably (1 × 10^6^ cells) were injected into the lateral tail vein for experimental metastasis model or fourth mammary pad for spontaneous metastasis model of 4-week-old female nude mice, respectively. The mice were divided randomly into 4 groups including Vehicle, SP2509 (25 mg/kg, i.p.), EPZ015666 (100 mg/kg, i.p.) and the combination of SP2509 and EZP015666 (five mice per group). The drugs were subsequently administered by intraperitoneal (i.p.) injection on days 10, 13, and 17 in an eight days repeating cycle for three cycles. The growth of primary tumors in mice that inoculated with cancer cells via fourth mammary fat pad injection were measured with a vernier caliper. The tumor volume was determined using the formula volume = (length) × (width)^2^ × 0.5. For the spontaneous metastasis assay, the lungs were removed and imaged using an IVIS Lumina II imaging system (Caliper life science, Hopkinton, MA) at day 41 post orthotopic transplantation. To examine for seeding metastases, mice that obtained cancer cells via intravenous injection were imaged at day 41 post tail veil injection by an IVIS Lumina II imaging system, then the lungs were removed and imaged using an IVIS Lumina II imaging system as well. All animal experiments were approved by the Animal Care Committee of Southern University of Science and Technology.

### Re-analysis of multiple ChIP-seq datasets

Three ChIP-seq raw datasets (GSE55421, GSE130194, and GSE101150) were derived from the GEO database (https://www.ncbi.nlm.nih.gov/geo/), using FASTQC (https://www.bioinformatics.babraham.ac.uk/projects/fastqc/) and Trim-galore (https://www.bioinformatics.babraham.ac.uk/projects/trim_galore/) to quality control and filtering for the sequencing reads. The sequencing reads were mapped to the reference genome (UCSC assembly hg38, GRCh38) through bowtie2 (version 2.4.5). The samtools were used for transforming the Sam format file to the Bam format file and to sort the Bam file. Using the samtools to remove the PCR duplicates, then Macs2 was applied for calculating the peak value of the ChIP-seq datasets to compare the treatment IP DNA and the control input DNA. A significant threshold of 10^–2^ was applied to all datasets. R-package ChIPseeker was used to annotate the peaks. We used MEME-ChIP for motif discovery. Using bamCoverage, we convert the bam file to the bw file. With the IGV genome browser, we visualize the peaks. The pie plot was drawn by the ggplot2 package and the Venn diagram was drawn by the Venn diagram package. All these packages were installed in the R (4.0.3).

### Dual-luciferase reporter assay

Wild-type (CCCAAA) or mutated (TCTGAG) E-cadherin or vimentin promoter sequence (-500 bp to TSS) was cloned into the firefly luciferase reporter pGL3-basic-based vector by homologous recombination with a ClonExpress® II One Step Cloning Kit (Vazyme, China). Next, HEK293T cells were co-transfected with luciferase reporter, pRL-TK Renilla luciferase vector and indicated expression constructs. After 48 h, the luciferase activity of total cell lysates was assayed using a Dual-Luciferase Reporter Assay System (Promega). Data was normalized against Renilla luciferase activity.

### Biotinylated oligonucleotide pull-down assay

Biotin-labeled double-stranded wild-type (CCCAAA) or mutated (TCTGAG) oligonucleotides or corresponding nonbiotinylated oligonucleotides were incubated with the Slug-PRMT5-LSD1 complex, which was prepared from HEK293T cells co-transfected with Flag-Slug, HA-PRMT5 and HA-LSD1 at 4 °C overnight under gentle rotation. The biotinylated DNA/protein complexes were captured using magnetic streptavidin beads at 4 °C for 2 h followed by three washes. Subsequently, bound proteins were detected by immunoblotting. Oligonucleotide sequences used were as follows: E-cadherin (wt): CAAAACGAACAAACAAAAAATCCCAAAAAACAAAAGAACTCAGCCAAGTG; vimentin (wt): TCAGACTATCATCCGGAAAGCCCCCAAAAGTCCCAGCCCAGCGCTGAAGT; E-cadherin (mut): CAAAACGAACAAACAAAAAATTCTGAG AAACAAAAGAACTCAGCCAAGTG; vimentin (mut): TCAGACTATCATCCGGAAAGCCTCTGAGAGTCCCAGCCCAGCGCTGAAGT;

### Statistical analysis

The data represent the mean ± SD values of samples obtained from three independent experiments. We performed a two-tailed Student’s t test to determine statistically significant differences between two groups. Differences between multiple groups were determined by Dunnett’s multiple comparison test. The Kaplan–Meier method was employed to plot survival curves, and differences were calculated with the log-rank test. The relationship between PRMT5 and LSD1 expression in human breast cancer samples were evaluated by χ^2^ test. In the statistical analysis, *P* < 0.05 is deemed as statistically significant.

## Results

### Slug interacts with PRMT5 and LSD1

**Fig. 1:**
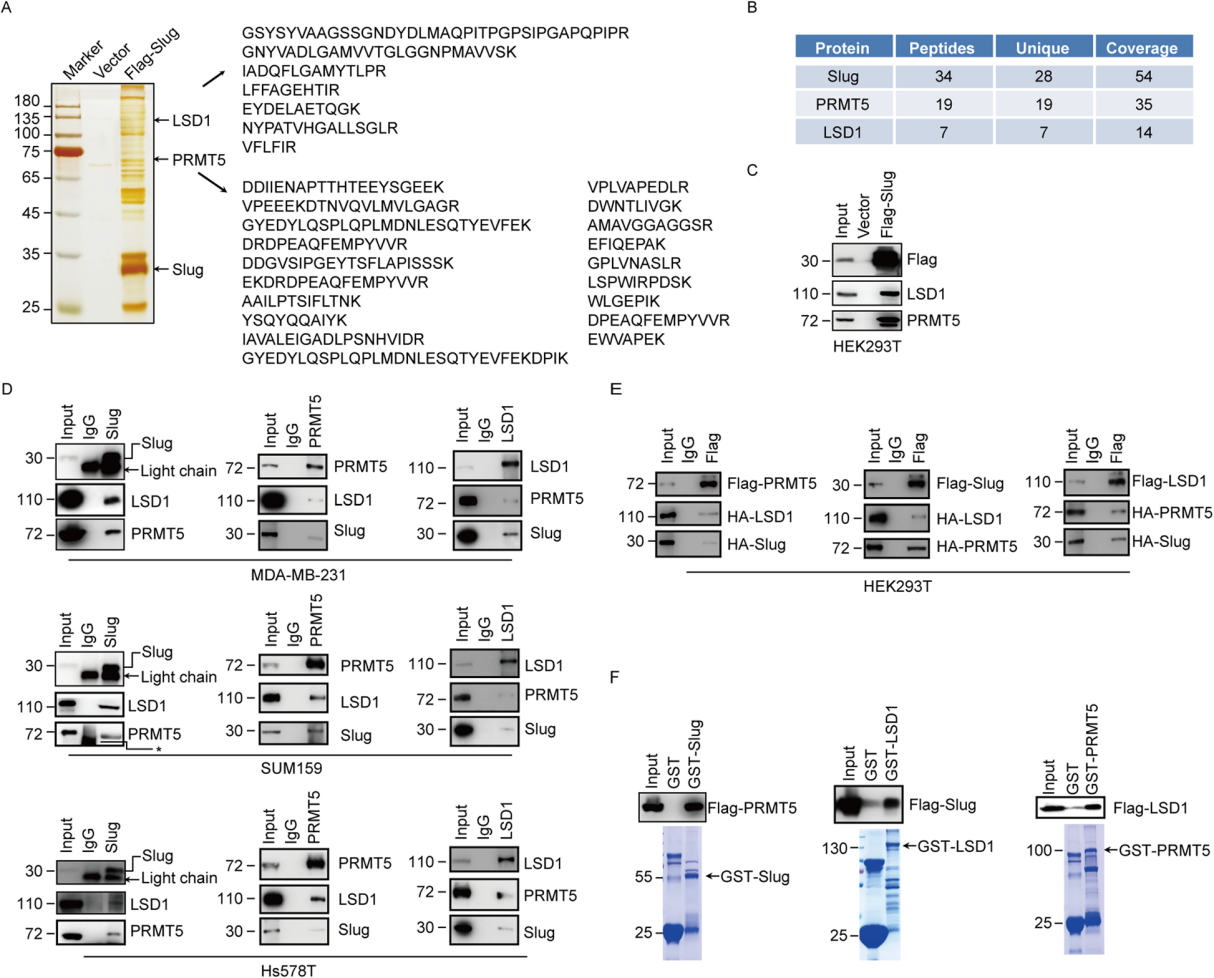
Slug interacts with PRMT5 and LSD1. (**A**) Immunoaffinity purification and mass spectrometry analysis of Slug-binding proteins. Extracts from HEK293T cells bearing Flag (Vector) or Flag-Slug were immunopurified with anti-Flag affinity columns and eluted with Flag peptide. The eluates were resolved by SDS-PAGE and visualized by silver staining. The protein bands on the gel were excised and identified by mass spectrometry. Representative peptide fragments of PRMT5 and LSD1 are indicated on the right. (**B**) Representative peptide coverage of the indicated proteins is shown in the table. (**C**) The purified fractions were analysed by western blotting with antibodies against indicated proteins. (**D**) Cell lysates from MDA-MB-231, SUM159 or Hs578T cells were immunoprecipitated with antibodies against indicated proteins followed by immunoblotting with various antibodies indicated. The arrows denote the light chains of IgG and Slug antibody. The asterisks indicate a nonspecific band. (**E**) Flag-PRMT5, HA-Slug and HA-LSD1 (Left), Flag-Slug, HA-PRMT5 and HA-LSD1 (Middle) or Flag-LSD1, HA-Slug and HA-PRMT5 (Right) were co-expressed in HEK293T cells, respectively. After immunoprecipitation with appropriate antibodies, bound proteins (e.g., Slug, PRMT5 or LSD1) were examined by western blotting. (**F**) GST-fused Slug, LSD1 or PRMT5 were incubated with the Flag-tagged PRMT5, Slug or LSD1 purified from HEK293T cells. The binding proteins by GST pull-down assays were examined by western blotting with indicated antibodies. Coomassie brilliant blue staining of the GST-fused proteins was shown below

To gain the mechanistic insight into the Slug mediated transcriptional regulation, we used the affinity purification and mass spectrometry coupled approach to survey the interactome of Slug. Whole cell extracts were prepared from HEK293T cells expressing Flag-Slug and subjected to purification using an anti-Flag affinity gel. Mass spectrometric analysis indicates that Slug immunoprecipitates contain LSD1 and PRMT5 (Fig. 1A, 1B). The presence of LSD1 and PRMT5 in the Slug interactome was validated by western blotting analysis of the column eluates with LSD1 and PRMT5 antibodies (Fig. 1C).

To ascertain the interactions among Slug, PRMT5 and LSD1, we performed the co-immunoprecipitation experiments with endogenous and exogenous proteins. We found that immunoprecipitation (IP) of endogenous Slug from MDA-MB-231, SUM159 and Hs578T cells brought down LSD1 and PRMT5, suggesting that Slug interacts with these proteins (Fig. 1D). Reciprocally, IP with antibodies against endogenous LSD1 or PRMT5, the remaining two proteins can also be detected (Fig. 1D). Moreover, we carried out the co-immunoprecipitation experiments in HEK293T cells expressing tagged Slug, PRMT5 or LSD1 as indicated in Fig. 1E, and detected the association among Slug, PRMT5 and LSD1. Consistent with these results, the Glutathione-S-transferase (GST) pull-down assay further supported the interactions among Slug, PRMT5 and LSD1 (Fig. 1F). Combined, these experiments indicate that Slug specifically interacts with PRMT5 and LSD1 in vivo.

### Molecular basis for the interactions among Slug, PRMT5 and LSD1

**Fig. 2:**
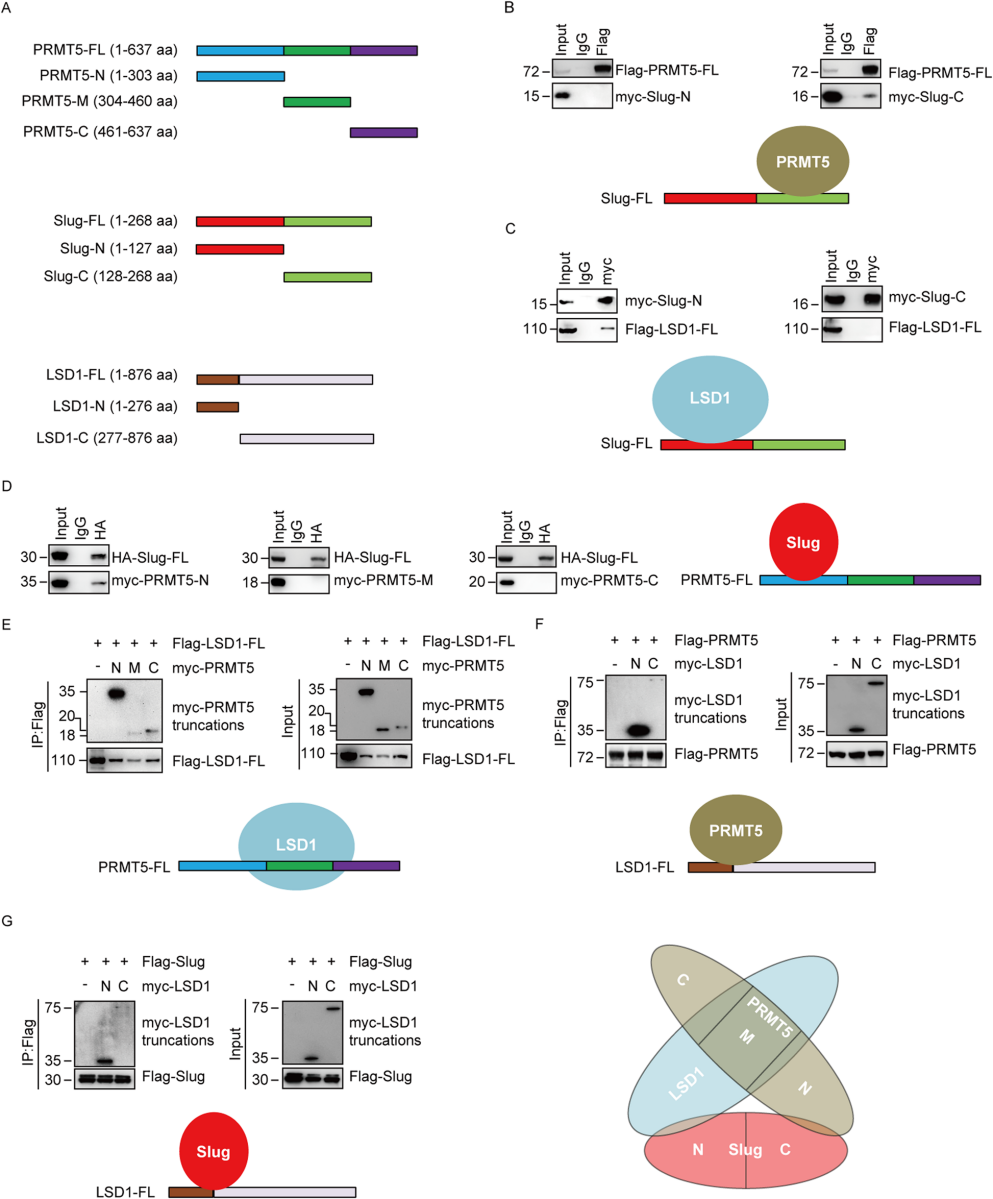
Molecular interaction between Slug, PRMT5 and LSD1. (**A**) Schematic diagram showing the structure of PRMT5, Slug and LSD1 and various deletion constructs used. (**B**) Mapping the domains of Slug required for the interaction with PRMT5. Flag-tagged full-length (FL) PRMT5 was co-expressed with Myc-tagged Slug truncations in HEK293T cells. Extracts were immunoprecipitated with Flag, bound proteins were examined by western blotting using myc. (**C**) Identification of the domains of Slug responsible for the interaction with LSD1. Flag-tagged LSD1-FL was co-expressed with Myc-tagged Slug truncations in HEK293T cells. After lysates were immunoprecipitated with Myc antibody, associated proteins were assessed by western blotting using Flag. (**D**) Mapping the domains of PRMT5 required for the interaction with Slug. HA-tagged Slug-FL was co-expressed with Myc-tagged PRMT5 truncations in HEK293T cells. Extracts were immunoprecipitated with HA, interacted proteins were examined by western blotting using Myc. (**E**) Identification of the domains of PRMT5 critical for the interaction with LSD1. Flag-tagged LSD1-FL was co-expressed with Myc-tagged PRMT5 truncations in HEK293T cells. Lysates were immunoprecipitated with Flag, associated proteins were examined by western blotting using Myc. (**F**) Mapping of the domains of LSD1 required for the interaction with PRMT5. Flag-tagged PRMT5-FL was co-expressed with Myc-tagged LSD1 truncations in HEK293T cells. Extracts were immunoprecipitated with Myc or Flag as indicated, bound proteins were examined by western blotting using Flag or Myc, respectively. (**G**) Identification of the domains of LSD1 important for the interaction with Slug. Flag-tagged Slug-FL was co-expressed with Myc-tagged LSD1 truncations in HEK293T cells. Extracts were immunoprecipitated with Flag, associated proteins were examined by western blotting using Myc. (**H**) Schematic diagram delineates the molecular interaction between Slug, PRMT5, and LSD1

To further delineate the interactions among Slug, PRMT5 and LSD1, a series of truncated mutants of these proteins were generated and transfected into HEK293T cells to map the domains critical for their association. The N-terminal Slug (1–127 aa) includes the SNAG domain of Slug, and the C-terminal Slug (128–268 aa) contains the conserved zinc finger motif (Fig. 2A). Expressing these two Slug deletions with full length PRMT5 (PRMT5-FL) or LSD1 (LSD1-FL) in HEK293T cells, we found that the C-terminal region of Slug is responsible for its interaction with PRMT5 (Fig. 2B), whereas the N-terminal region of Slug associates with LSD1 (Fig. 2C).

PRMT5 contains three functional domains: the N-terminal TIM barrel region (1–303 aa), the middle Rossmann-fold segment (304–460 aa) and the C-terminal β-barrel domain (461–637 aa) (Fig. 2A). To identify the region responsible for the PRMT5 interaction with Slug or LSD1, we generated various PRMT5 domain-deletion mutants and expressed them with Slug or LSD1 in HEK293T cells. The N-terminal truncation of PRMT5 retained the ability to bind Slug (Fig. 2D). Interestingly, all PRMT5 mutants interact with LSD1, while the N-terminal-truncated PRMT5 are mostly responsible for interaction with LSD1 (Fig. 2E).

The N-terminal region of LSD1 comprises a SWIRM domain (1–276 aa) and the larger C-terminal segment of LSD1 includes a catalytic amine oxidase (AO) domain (277–876 aa) (Fig. 2A). The domain deletion mutants of LSD1 were co-transfected with Slug or PRMT5 into HEK293T cells. Co-IP analysis demonstrated that both the N-terminal and C-terminal region of LSD1 were able to interact with PRMT5 or Slug, whereas the interaction of the C terminus of LSD1 and PRMT5 (Fig. 2F) or Slug (Fig. 2G) was less significant. Collectively, our findings further support the specific associations among Slug, PRMT5 and LSD1, and provide the details of the molecular interactions relevant for the formation of the Slug-PRMT5-LSD1 complex, as schematically summarized in Fig. 2H.

### Genome-wide analysis of transcriptional targets for Slug and its associated proteins LSD1 and PRMT5

**Fig. 3:**
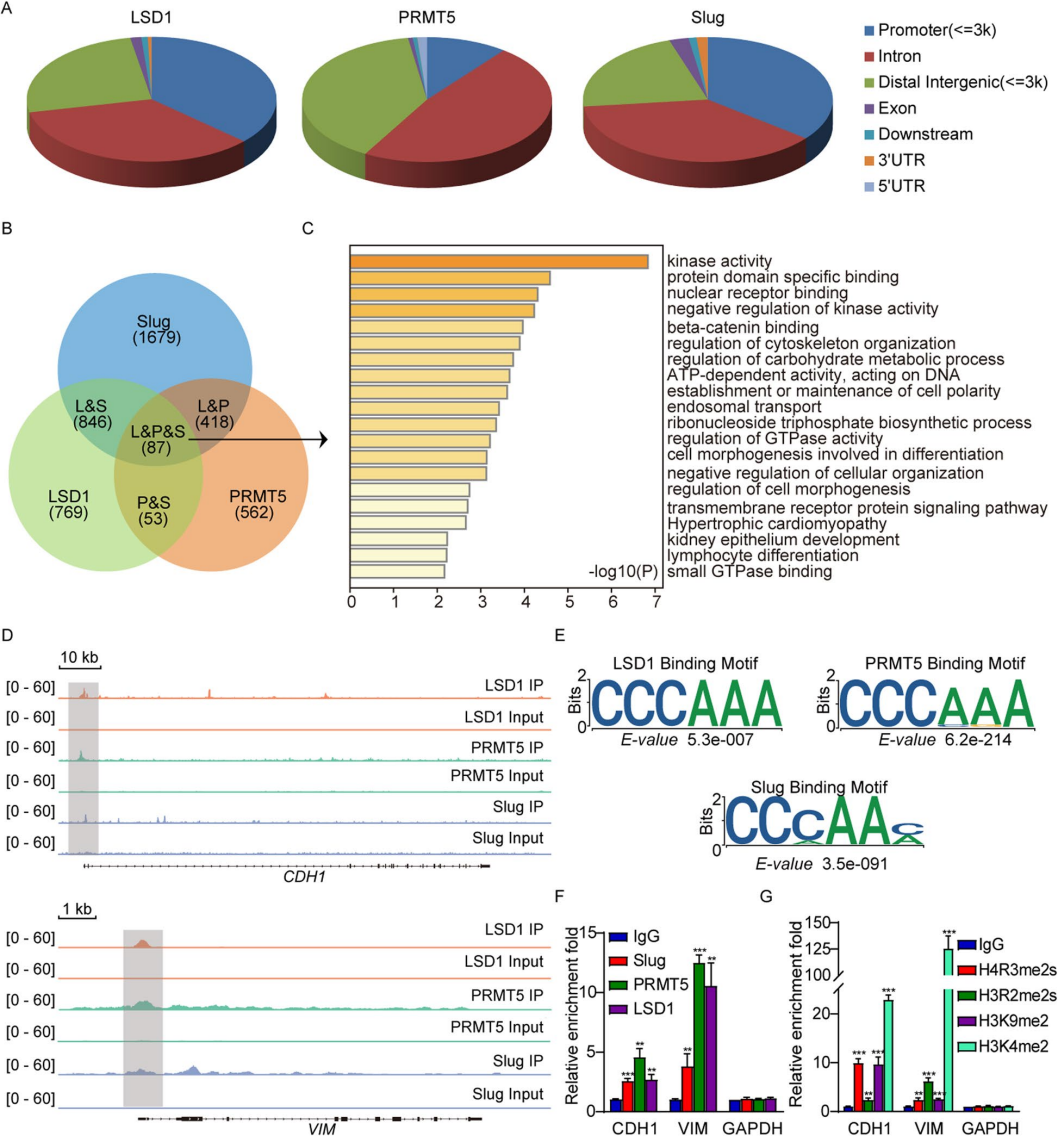
Genome-wide transcription target analysis of the Slug-PRMT5-LSD1 complex. (**A**) Public ChIP-seq datasets (GSE55421, GSE130194 and GSE101150) were extracted from Gene Expression Omnibus (GEO) database for genome-wide identification of the Slug, PRMT5 and LSD1 binding sites. The percentages of binding sites mapped to seven different genomic regions are shown in the periphery of the pie chart. (**B**) Venn diagram of overlapping promoters bound by Slug, PRMT5 and LSD1. The number of genes targeted by themselves is indicated. (**C**) Gene ontology (GO) analysis of the 87 overlapped target genes is shown. Based on the different functions of these genes, the GO function network was built (Left). Barplots represent top20 GO Biological Processes, ranked by − log10 (p.value) (Right) (**D**) Visualized binding peaks of Slug, PRMT5 and LSD1 at representative target genes (CDH1 and VIM) loci using a genome browser (IGV). (**E**) The binding motifs for Slug, PRMT5 and LSD1 were analyzed by MEME suite. (**F**) Verification of the ChIP-seq results by qChIP analysis of the indicated genes in MDA-MB-231 cells. The occupation of Slug, PRMT5 and LSD1 at the indicated promoters in MDA-MB-231 cells was analyzed with the qChIP assay. (**G**) The level of H4R3me2s, H3R2me2s, H3K9me2 and H3K4me2 at the indicated promoters in MDA-MB-231 cells was analyzed with the qChIP assay. For E and F, results are represented as the fold-change compared to the control IgG. Error bars represent the mean ± SD from three independent experiments (**p < 0.01, ***p < 0.001, and two-tailed unpaired t-test)

To unravel the function and significance of the association among Slug, PRMT5 and LSD1, we analyzed the genome-wide transcriptional targets of the Slug-LSD1-PRMT5 complex by mining previously published ChIP-Seq datasets of Slug (GSE55421), PRMT5 (GSE130194) and LSD1 (GSE101150). We identified 7136 LSD1-specific binding peaks, 19,628 PRMT5-specific binding sites, and 73,702 Slug-specific binding sequences mostly residing in the promoter, intronic or intergenic regions (Fig. 3A, q (FDR) value cut off of 0.05). The data were then cross-analyzed for overlapping binding sites at the promoters for potential co-targets of Slug, PRMT5 and LSD1. A total of 87 specific promoters targeted by Slug, PRMT5 and LSD1 were identified (Fig. 3B). Gene ontology (GO) analysis with Metascape online analysis tool (https://metascape.org/) was applied to uncover various cellular events for the genes corresponding to these co-occupied promoters. These biological processes include cytoskeleton organization, cell morphogenesis, metabolism and development (Fig. 3C).

Importantly, Slug, PRMT5 and LSD1 exhibited similar peaks on the proximal promoter region of the EMT genes such as E-cadherin (CDH1) and vimentin (VIM) (Fig. 3D). Analysis of the genomic distributions of Slug, PRMT5 and LSD1 revealed similar binding motifs (Fig. 3E), suggesting that these proteins are functionally connected. Quantitative ChIP (qChIP) analysis in MDA-MB-231 cells with the antibodies against Slug, PRMT5, LSD1 on selected genes (e.g., CDH1 and VIM) showed that the promoters of these two genes were strongly enriched (Fig. 3F), validating the results derived from the public ChIP-seq datasets. In addition, qChIP analysis with the antibodies against H3K4me2 and H3K9me2 (two LSD1 substrates), H4R3me2s and H3R2me2s (two PRMT5 targets) revealed that the target promoters of CDH1 and VIM were specifically marked with H3K4me2, H3K9me2, H4R3me2s and H3R2me2s (Fig. 3G), further supporting the occupancy of these promoters by PRMT5 or LSD1.

### Transcription regulation of CDH1 and VIM by the Slug-PRMT5-LSD1 complex

**Fig. 4:**
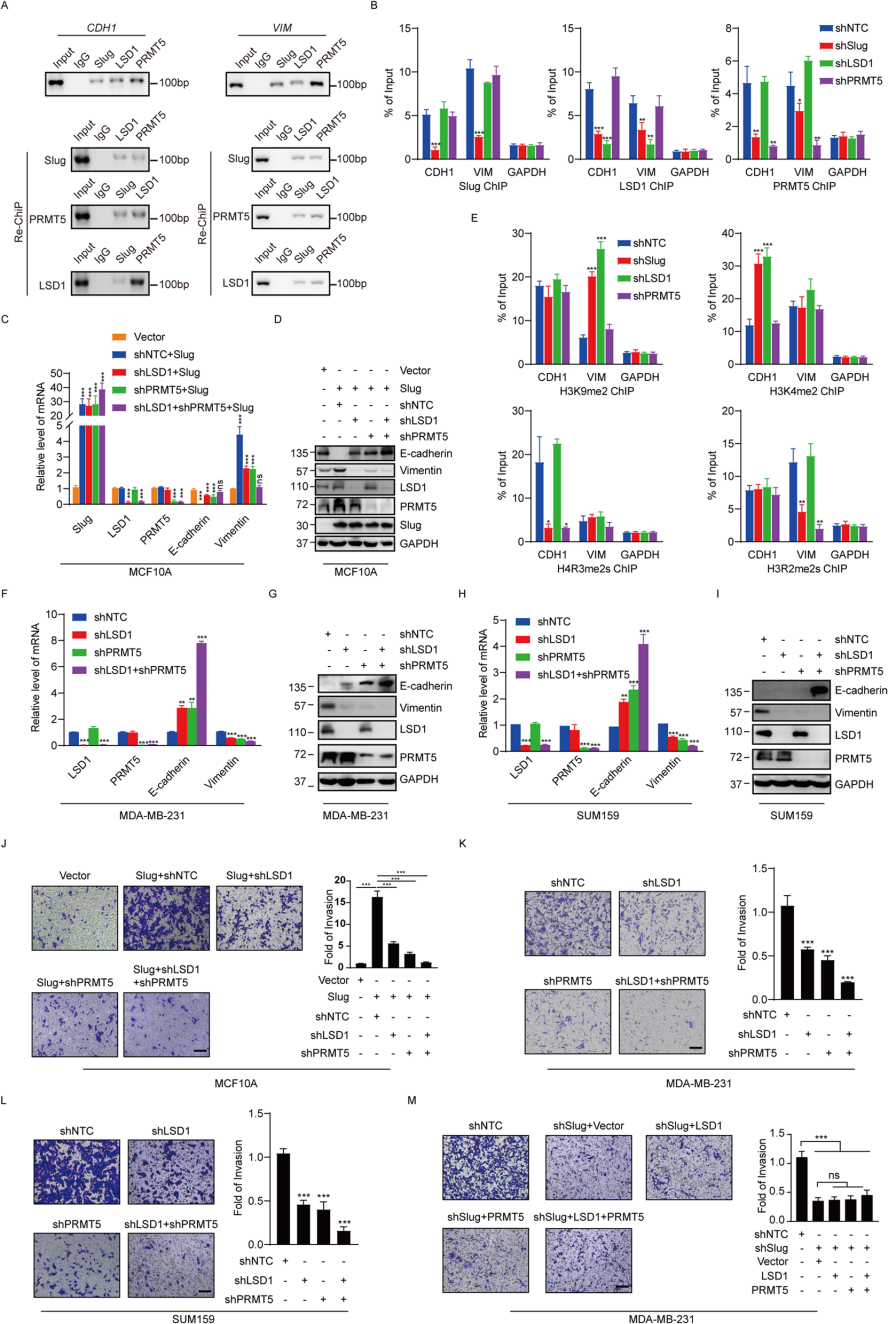
Slug cooperates with PRMT5 and LSD1 to regulate the transcription of E-cadherin and vimentin. (**A**) ChIP and Re-ChIP experiments were done in MDA-MB-231 cells with the indicated antibodies. (**B**) MDA-MB-231 cells were infected with lentiviruses bearing the indicated shRNAs, and the association of Slug, PRMT5, LSD1 at the promoter of E-cadherin and vimentin was analyzed with the qChIP assay. (**C** and **D**) MCF10A cells were infected with lentiviruses carrying the indicated shRNAs together with Slug expression constructs or empty vector. The mRNA or protein level of indicated genes was monitored by qPCR (**C**) or western blotting (**D**). (**E**) MDA-MB-231 cells were infected with lentiviruses carrying the indicated shRNAs, and the association of H4R3me2s, H3R2me2s, H3K9me2 and H3K4me2 at the promoter of E-cadherin and vimentin was analyzed with the qChIP assay. (**F** and **G**) MDA-MB-231 cells were infected with lentiviruses carrying shNTC, shLSD1, shPRMT5 and shLSD1 + shPRMT5. The mRNA or protein level of indicated genes was measured by qPCR (**F**) or western blotting (**G**). (**H** and **I**) SUM159 cells were infected with lentiviruses carrying shNTC, shLSD1, shPRMT5 and shLSD1 + shPRMT5. The mRNA or protein level of indicated genes was measured by qPCR (**H**) or western blotting (**I**). (**J**) MCF10A cells were infected with lentiviruses carrying the indicated shRNAs together with Slug expression constructs or empty vector. The invasiveness of these cells was analyzed with a matrigel-coated chamber invasion assay. (**K** and **L**) MDA-MB-231 (**K**) or SUM159 (**L**) cells were infected with lentiviruses carrying shNTC, shLSD1, shPRMT5 and shLSD1 + shPRMT5. (**M**) MDA-MB-231 cells transfected with shSlug and the expression plasmids for PRMT5 or LSD1 for cell invasion assay. For J, K, L and M, representative photomicrographs are shown in the left. The histograms show the mean ± SD of the fold changes of numbers of invasive cells in each of samples, relative to that of control sample from three separate experiments. ***P < 0.001, as determined by Student’s t test. For B, C, E, F and H, the data represent the mean ± SD from three independent experiments (**p < 0.01, ***p < 0.001, Student’s t-test)

We then evaluated the regulation of E-cadherin and vimentin by the Slug-PRMT5-LSD1 complex. We found that Slug, PRMT5 and LSD1 co-occupied the promoters of E-cadherin and vimentin through the ChIP assays using antibodies against Slug, PRMT5 or LSD1 in MDA-MB-231 cells (Fig. 4A, upper panel). To further evaluated the conjecture that Slug, PRMT5 and LSD1 function in the same protein complex at the E-cadherin and vimentin promoters, ChIP/Re-ChIP experiments were carried out on the representative target gene CDH1 and VIM promoters in MDA-MB-231 cells. Soluble chromatin was first immunoprecipitated with antibodies against Slug, PRMT5 or LSD1. The immunoprecipitates were subsequently re-immunoprecipitated with indicated antibodies. The results demonstrated that the CDH1 and VIM promoters initially immunoprecipitated with antibodies against Slug could be re-immunoprecipitated with antibodies against LSD1 or PRMT5 (Fig. 4A, lower panel). Similar results were obtained when an initial ChIP was performed with antibodies against LSD1 or PRMT5 (Fig. 4A). These results support that Slug, PRMT5 and LSD1 occupy the target promoters together.

To further confirm the transcription regulation of E-cadherin/vimentin by the Slug/PRMT5/LSD1 complex, we performed dual-luciferase reporter assay. As shown, Slug was able to repress the E-cadherin (WT) or activate vimentin (WT) promoter activity (Figure S1A). The mutated promoters did not respond to Slug (Figure S1A). Consistently, Slug was no longer able to repress the E-cadherin or activate vimentin promoter activity when PRMT5 or LSD1 was silenced, further supporting the targeting of E-cadherin and vimentin by the Slug/PRMT5/LSD1 complex (Figure S1A). To test the binding specificity, we mutated the binding motif CCCAAA (WT) to TCTGAG (Mut) and performed biotinylated oligonucleotide pull-down assay. We determined that Slug, PRMT5 or LSD1 specifically binds to the wild-type, but not to the mutant E-cadherin or vimentin probes (Figure S1B). Taken together, we concluded that Slug/PRMT5/LSD1 binds directly to CCCAAA motif in the E-cadherin and vimentin promoters.

We wondered how Slug, PRMT5 and LSD1 are recruited to the target genes. MDA-MB-231 cells were infected with shRNAs targeted to Slug, PRMT5 and LSD1 mRNA along with a shNTC control. The knockdown effects of shRNAs were confirmed by Western blotting (Figure S2). Q-ChIP experiments indicate that the depletion of Slug, PRMT5 or LSD1 led to a drastic reduction of the recruitment of the corresponding protein to the target promoters of CDH1 and VIM (Fig. 4B). Interestingly, whereas the Slug knockdown was associated with a reduced recruitment of PRMT5 and LSD1 on the CDH1 and VIM promoters, the depletion of either PRMT5 or LSD1 had only negligible effect on the recruitment of Slug (Fig. 4B), suggesting that PRMT5 and LSD1 are recruited on target promoters by Slug to act as transcription regulators.

Among the identified target genes of the Slug-PMRT5-LSD1 complex, CDH1 and VIM are important molecular markers of EMT. Downregulation of epithelial cell markers, like E-cadherin, and enhanced expression of mesenchymal markers such as vimentin, have been characterized as hallmarks during EMT process. To examine the transcription repression of CDH1 and transcription activation of VIM by the Slug-PRMT5-LSD1 complex, Slug was overexpressed in MCF10A cells, leading to decreased expression of E-cadherin and increased expression of vimentin at both the transcriptional and protein levels (Fig. 4C, 4D and Figure S3). Significantly, the alterations of E-cadherin and vimentin upon Slug overexpression were offset when PRMT5 or LSD1 was depleted in MCF10A cells, and this weakening trend was even more pronounced when LSD1 and PRMT5 were simultaneously knocked down (Fig. 4C, 4D and Figure S3). Since the promoter recruitment of Slug, PRMT5 and LSD1 is consistent with the E-cadherin and vimentin expression patterns, it appears that Slug functions in a dual mode in modulating gene expression during the EMT process.

To further gain the molecular insights into the dual regulatory mode mediated by the Slug-PRMT5-LSD1 complex on the E-cadherin and vimentin promoters, the expression of Slug, PRMT5 or LSD1 was individually silenced by their corresponding shRNA in MDA-MB-231 cells. Subsequent qChIP experiments showed that a marked increase in H3K9me2 but largely unchanged H3K4me2 on the vimentin promoter upon the depletion of Slug or LSD1, as well as a significant decrease of H3R2me2s but unchanged H4R3me2s on the vimentin promoter upon Slug or PRMT5 depletion (Fig. 4E). On the other hand, the qChIP analysis revealed that the levels of H3K4me2 were markedly increased at the E-cadherin promoter upon the depletion of Slug or LSD1, and the levels of H4R3me2s were significantly reduced at the E-cadherin promoter upon Slug or PRMT5 knockdown, whereas the levels of H3K9me2 and H3R2me2s did not change much upon knockdown of Slug, LSD1, or PRMT5 individually (Fig. 4E). Collectively, these experiments indicate that PRMT5 and LSD1 were recruited by Slug to suppress E-cadherin expression and activate vimentin transcription. Moreover, co-silencing of PRMT5 and LSD1 resulted in more prominent changes than individual knockdowns in the expression of the two EMT markers in MDA-MB-231 (Fig. 4F and G and Figure S3) and SUM159 (Fig. 4H and I and Figure S3) cells. These results further support the notion that Slug coordinates with PRMT5 and LSD1 to orchestrate the transcription of E-cadherin and vimentin. Previous studies have revealed that Slug is able to transcriptionally inhibit Claudin1 and transcriptionally activate ZEB1 [11, 12]. We found that the Slug-PRMT5-LSD1 complex mediated dual regulatory mode is also adapted to Claudin-1 and ZEB1 genes modulation (Figure S4A-C).

Given the roles of Slug, LSD1 and PRMT5 in EMT and cancer progression, we explored the functional coordination of the Slug-PRMT5-LSD1 complex in cell invasion by the transwell assay. Consistent with aforementioned the functional link between Slug, LSD1 and PRMT5, the positive effect of Slug overexpression on the invasive ability of MCF10A cells was partially attenuated by LSD1 or PRMT5 knockdown, and more severely reduced upon simultaneous depletion of PRMT5 and LSD1 (Fig. 4J). In addition, in the highly invasive MDA-MB-231 and SUM159 cells, the depletion of PRMT5 or LSD1 separately resulted in decreased invasive potential of these cells, and co-knockdown of PRMT5 and LSD1 led to more pronounced reduction in the cell invasion (Fig. 4K, 4L). Moreover, MDA-MB-231 with Slug depletion led to a decrease in the invasive potential, whereas the inhibitory effect of Slug knockdown on the invasiveness was not significantly rescued when PRMT5 or LSD1 was ectopically expressed in MDA-MB-231 cells (Fig. 4M). Taken together, these data support a vital role for the Slug-PRMT5-LSD1 complex in the regulation of invasion.

### Co-inhibition of PRMT5 and LSD1 synergistically suppresses breast cancer progression

**Fig. 5:**
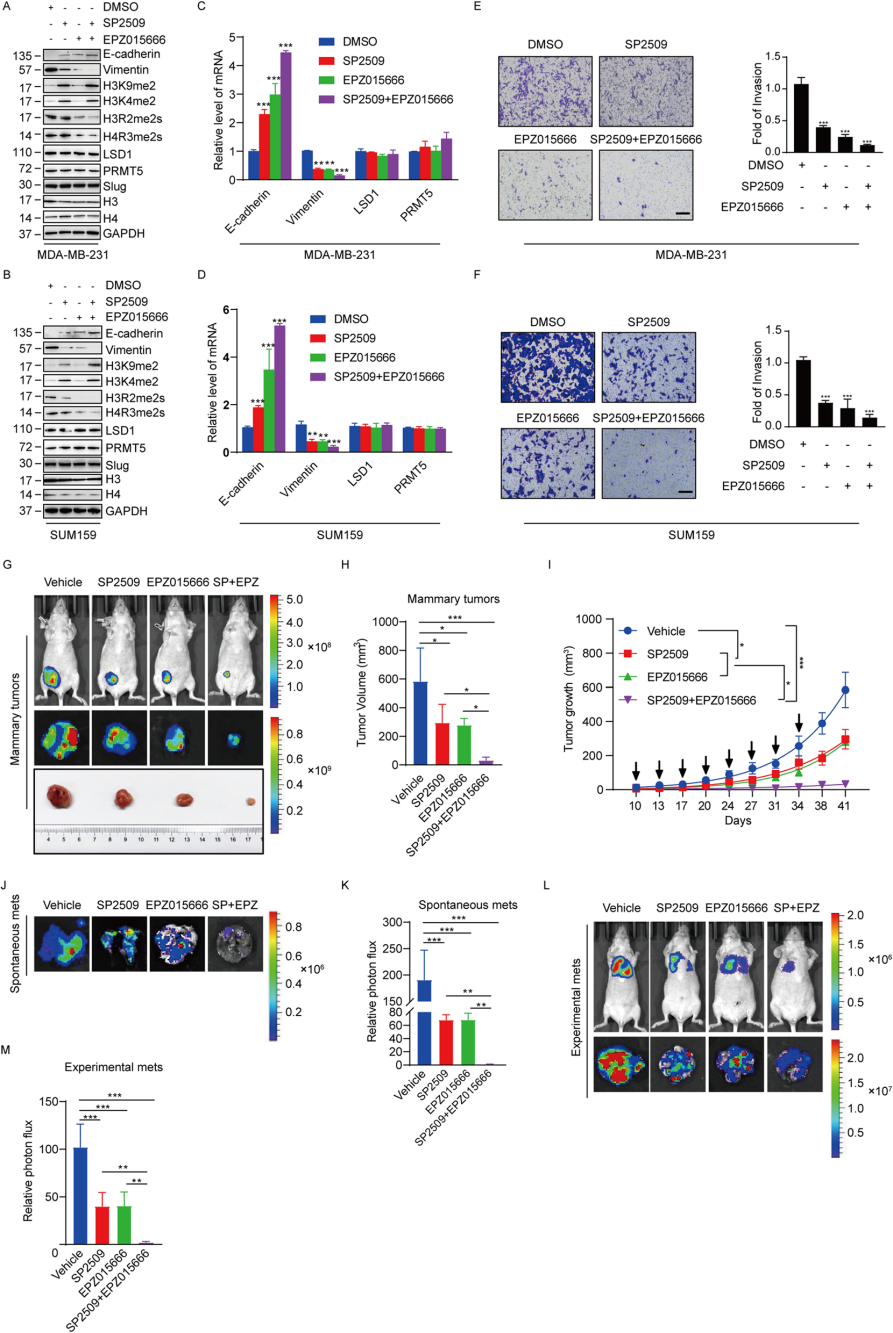
Combination of SP2509 and EPZ015666 synergistically inhibits tumor progression of breast cancer cells (**A** and **B**) Immunoblot analysis of the indicated proteins in MDA-MB-231 (**A**) and SUM159 (**B**) cells treated with DMSO, SP2509 (1 μM), EPZ015666 (1 μM) or SP2509 plus EPZ015666 for 72 h. (**C** and **D**) Expression of the indicated genes was measured by qPCR in MDA-MB-231 (**C**) and SUM159 (**D**) cells treated with DMSO, SP2509 (1 μM), EPZ015666 (1 μM) or SP2509 plus EPZ015666 for 72 h. (**E** and **F**) MDA-MB-231 (**E**) and SUM159 (**F**) cells treated with DMSO, SP2509 (1 μM), EPZ015666 (1 μM) or SP2509 plus EPZ015666 for 72 h. The invasiveness of these cells was analyzed with a matrigel-coated chamber invasion assay. Representative photomicrographs are shown in the left. The histograms show the mean ± SD of the fold changes of numbers of invasive cells in each sample over that of control sample from three independent experiments. ***P < 0.001, as determined by Student’s t test. (**G**) MDA-MB-231 cells stable expressing firefly luciferase were inoculated orthotopically into the fourth mammary fat pad of 6-week-old female nude mice. The mice were treated with Vehicle, SP2509 (25 mg/kg, i.p.), EPZ015666 (100 mg/kg, i.p.) and combination of SP2509 and EZP015666 (*n* = 5) starting from day 10 post transplantation. The drugs were administered on days 10, 13, and 17 in an eight days repeating cycle for three cycles. Primary tumors were quantified by bioluminescence imaging on day 41 after initial implantation. Representative in vivo (upper) and in vitro (middle) tumor bioluminescent images, and excised tumors (lower) from each of group are shown. (**H**) The bar graphs show the mean ± SD of the primary tumor volume from mice in G. (**I**) The graph depicts the mean tumor growth of mice in G which received indicated treatment on the days marked by the black arrows. (**J**) Representative ex vivo bioluminescence imaging of the lungs removed from mice in G on day 41. (**K**) The bar graphs show the mean ± SD ex vivo lung photon flux of mice in G for each group. (**L**) MDA-MB-231 cells stable expressing firefly luciferase were intravenously injected into female nude mice. The mice were treated as described in G. Lung metastasis was quantified using bioluminescence imaging on day 41 after initial implantation. Representative in vivo (upper) and ex vivo lungs (lower) bioluminescence imaging from each of group are shown. (**M**) The bar graphs indicate the mean ± SD ex vivo lung photon flux of mice in L for each group. For H, I, K and M, *p < 0.05, ***P < 0.001, Dunnett’s multiple comparison test

Despite the crucial role of Slug in modulating EMT and breast cancer metastasis, there is no effective method to directly target Slug pharmaceutically. Since PRMT5 and LSD1 are epigenetic enzymes that are more druggable than Slug itself, combined targeting of PRMT5 and LSD1 may be a more effective therapeutic strategy for the treatment of metastatic breast cancer. To investigate whether the combined inhibition of PRMT5 and LSD1 would synergistically impede breast cancer progression, we employed a PRMT5 inhibitory compound EPZ015666 and a selective LSD1 inhibitor SP2509. We found that SP2509 treatment effectively increased the level of H3K4me2 and H3K9me2 in MDA-MB-231 and SUM159 cells, without affecting the levels of H4R3me2s and H3R2me2s (Fig. 5A, B). In contrast, EPZ015666 treatment effectively decreased the levels of H4R3me2s and H3R2me2s, but did not alter the level of H3K4me2 and H3K9me2 (Fig. 5A, B) in MDA-MB-231 and SUM159 cells. Moreover, the two inhibitors did not affect the Slug, PRMT5 and LSD1 protein levels overall (Fig. 5A, B) and the interaction among them (Figure S5A). These results suggest that SP2509 and EPZ015666 effectively impaired the enzymatic activity of LSD1 and PRMT5 respectively in breast cancer cells.

The effects of EPZ015666 and SP2509 separately or combined on EMT of breast cancer cells were assessed by western blotting and qPCR. The results revealed that either EPZ015666 or SP2509 resulted in the induction of E-cadherin and the reduction of vimentin, and strikingly the treatment of both EPZ015666 and SP2509 led to stronger changes than single inhibitor treatment in the expression of these two markers at both protein (Fig. 5A, B and Figure S5B) and mRNA (Fig. 5C, D and Figure S5B) levels in breast cancer cells. In line with these findings, the treatment of SP2509 or EPZ015666 alone decreased breast cancer cell invasion potential, whereas the double inhibitor treatment led to a significantly stronger inhibitory effect (Fig. 5E, F).

We next evaluated the effects of EPZ015666 and SP2509 on breast tumor growth and metastasis in vivo. MDA-MB-231 cells stably expressing firefly luciferase were orthotopically implanted onto female nude mice mammary fat pat or intravenously injected into female nude mice for the study of spontaneous metastasis or seeding lung metastasis, respectively. After 10 days, the mice xenografted with breast cancer tumors were then divided into a control group and various treatment groups, including the SP2509 group (25 mg/kg), the EPZ015666 (100 mg/kg) group, the SP2509 plus EPZ015666 group. Tumor volumes were measured at indicated time with calipers and harvested on day 41. Monotherapy with either SP2509 or EPZ015666 alone partially inhibited the growth of the breast tumors, interestingly, the combined addition of SP2509 and EPZ015666 caused obvious synergistic effects in reducing tumor volumes (Fig. 5G-I). Moreover, the results revealed that, in the orthotopically implanted groups, the combination therapy with SP2509 and EPZ015666 was significantly more effective in decreasing spontaneous lung metastasis than monotherapy with either SP2509 or EPZ015666 alone (Fig. 5J, K). In addition, in the intravenous groups, the treatment with both drugs together resulted in a more dramatic decrease in experimental lung metastasis than the treatment with either inhibitor alone (Fig. 5L, M). Furthermore, we did not observe obvious drug toxicity during the course of treatment on mice, suggesting dosage and therapeutic regimen were well tolerated in mice (Figure S5C). Taken together, these data demonstrate that targeting both PRMT5 and LSD1 for inhibition is a potential novel therapeutic option for metastatic breast cancer patients.

### PRMT5 and LSD1 are coordinately expressed in breast tumor specimens and their high expression portends poor prognosis in breast cancer patients

**Fig. 6:**
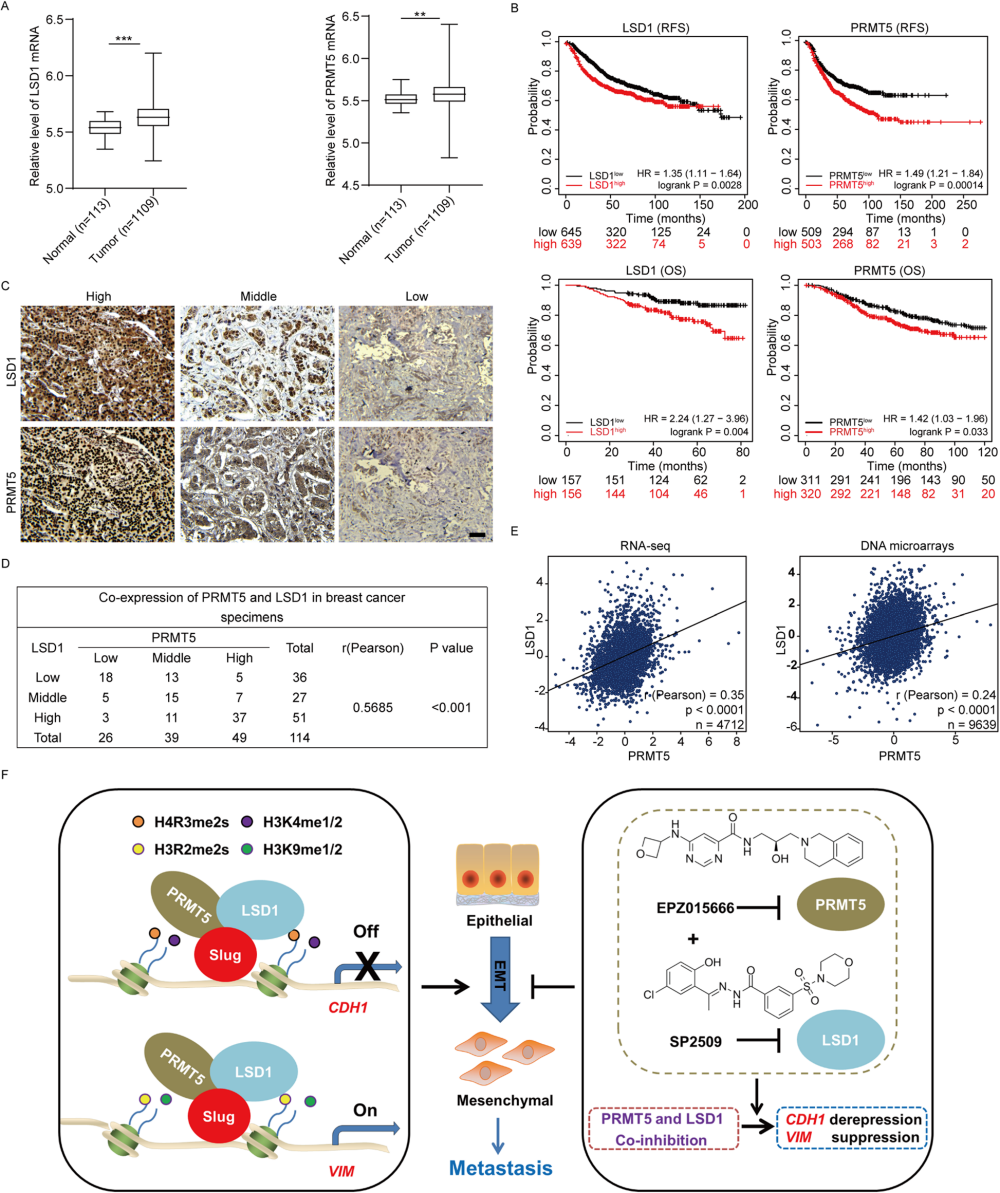
Expression of PRMT5 and LSD1 are positively associated in breast cancer patients (**A**) LSD1 (left) and PRMT5 (right) mRNA expression levels in normal (*n* = 113) and breast cancer tissues (*n* = 1109) in the TCGA microarray database. (**B**) Kaplan–Meier survival analysis of the relationship between relapse-free survival (upper), overall survival (lower) and the expression LSD1 (left) or PRMT5 (right), Statistical significance was determined by log-rank test. (**C**) Representative immunohistochemical labeling of LSD1 and PRMT5 (brown) in different breast cancer specimens with high, middle and low expression of PRMT5 and LSD1 as indicated. The nuclei were counterstained with hematoxylin (blue). Scale bars = 50 μm. (**D**) The relationship between LSD1 and PRMT5 expression in breast cancers was assessed by χ^2^ analysis, and the strength of correlation was evaluated by Pearson correlation coefficient. Statistical significance was defined as *p* < 0.05. (**E**) Analysis of public clinical datasets for the expression of PRMT5 and LSD1 in an RNA-sequencing dataset (left) and a DNA microarray dataset (right). The relative mRNA level of LSD1 was plotted against that of PRMT5. Correlations were analyzed using Pearson correlation method. (**F**) A proposed model illustrates the dual mode of transcriptional regulation of Slug conferred by PRMT5 and LSD1, leading to an EMT and metastasis and pharmacologic co-inhibition of PRMT5 and LSD1 synergistically suppresses breast cancer progression (see Discussion)

As the combined targeting of PRMT5 and LSD1 presents an effective approach against metastatic breast cancer, we extended our analysis to a clinically and pathologically relevant context. We therefore surveyed publicly available gene-expression data in The Cancer Genome Atlas (TCGA) database to compare PRMT5 or LSD1 expression in normal human breast tissues and breast cancer specimens. We found that breast cancer samples expressed significantly higher PRMT5 or LSD1 levels than normal breast cells, (*n* = 1222, *P* < 0.01, Fig. 6A). Next, Kaplan–Meier survival analysis with online tool (http://kmplot.com/analysis/) demonstrated that both enhanced LSD1 expression and higher PRMT5 expression were associated with shorter relapse-free survival (RFS) and overall survival (OS) of breast cancer patients (Fig. 6B). These data suggest that the enhanced expression of PRMT5 and LSD1 is associated with adverse outcomes of breast cancer patients.

If the combined targeting of PRMT5 and LSD1 is clinically meaningful, then PRMT5 and LSD1 likely would exhibit similar expression pattern in breast cancer patients. To this end, we first test the correlation between the expression level of PRMT5 and LSD1 in 114 human breast cancer samples using immunohistochemical (IHC) analysis. Interestingly, the expression of PRMT5 positively correlated with LSD1 in breast tumor specimens (Fig. 6C, D). To further ascertain this finding, we analyzed publicly available gene-expression datasets that have larger sample size of breast cancer patients. The results showed that PRMT5 expression was significantly positively correlated with the level of LSD1 in both RNA-sequencing dataset (*n* = 4712, *P* < 0.001, Fig. 6E, left) and DNA microarray dataset (*n* = 9639, *P* < 0.001, Fig. 6E, right).

We further analyzed the PRMT5 and LSD1 co-expression in distinct breast cancer subtypes. We found that PRMT5 expression was positively correlated with the expression of LSD1 in both mRNA and protein level among all subtypes including luminal, HER2 + and Basal-like subtype (Figure S6A, B). Interestingly, we noticed that this kind of positive correlation trend seemed to be a little bit more pronounced in basal-like breast patients with higher malignancy (Figure S6A, B). Collectively, these data support the observation that expression pattern of PRMT5 and LSD1 is similar in breast cancer patients.

## Discussion

In this study, we report that Slug, PRMT5 and LSD1 interact and work together to modulate the expression of E-cadherin and vimentin that are the molecular markers of EMT. Slug has been documented as both an activator and a repressor of transcription on different target genes involved in EMT program [9–12]. The molecular basis underlying this dual mode of transcriptional regulation remains elusive. We found that Slug interacts with PRMT5 and LSD1 and is required for their recruitment to the E-cadherin promoter to inhibit E-cadherin expression by PRMT5-catalyzed H4R3me2s and LSD1-mediated demethylation of H3K4me2 on the E-cadherin promoter. On the other hand, Slug brings PRMT5 and LSD1 to the vimentin promoter to function as a transcriptional activator to promote vimentin expression via catalyzing H3R2me2s by PRMT5 and removing H3K9me2 by LSD1 respectively on the vimentin promoter. Importantly, combined inhibition of PRMT5 and LSD1 synergistically impedes the EMT and breast cancer progression (Fig. 6F).

PRMT5 is known to interact with several transcription complexes to formulate a transcription supression program to promote EMT process. Previous studies identified PRMT5 as a suppressor recruited to the Snail complex through the interplay with NuRD (MTA1) or AJUBA corepressor to inhibit the expression of a group of genes such as E-cadherin and α-catenin [23, 24]. PRMT5 also interacts with c-MYC, which in turn is critical for H4R3me2s in repressing target genes to promote gastric cancer progression [25].

LSD1 has been suggested to coordinate with EMT transcription factors and chromatin-modifying enzymes to act as a transcription repressor to trigger the EMT program. It is well documented that Snail brings the LSD1-CoREST complex to the E-cadherin promoter for transcriptional repression by catalyzing the demethylation of H3K4me2 [16]. This is consistent with our finding that Slug recruits PRMT5 and LSD1 to the promoter of E-cadherin to transcriptionally repress the E-cadherin expression. However, our study shows that the Slug-PRMT5-LSD1 complex is able to not only transcriptionally inhibit E-cadherin expression, but also activate Vimentin expression. PRMT5-driven H3R2me2s is usually localized in the euchromatic region and associated with transcription activation [26, 27]. PRMT5 was reported to bind CRTC2 and is recruited to the promoters of gluconeogenic genes to activate the transcription of gluconeogenic genes upon glucagon stimulation [28]. Another study has shown that OXR1A interacts with PRMT5 and facilitates PRMT5-mediated H3R2me2s on the Gh promoter to increases its transcription in the pituitary gland [29]. Besides H3K4 demethylation, LSD1 is also able to mediate H3K9me2 demethylation, leading to transcriptional activation. For instance, LSD1 associates with the Androgen Receptor (AR) to trigger the transcription of a group of AR target genes by LSD1-driven removal of H3K9me2 [30]. LSD1 is required for inducing a series of ERα-regulated genes through H3K9me2 demethylation at the promoter and enhancer regions in response to estrogens stimulation [31]. This dual function of PRMT5 or LSD1 in both transcription activation and repression has been well studied. Combined, it is likely that the dual mode of Slug in transcription regulation during the EMT process is likely conferred by its coordination with PRMT5 and LSD1.

Our study indicates that PRMT5 and LSD1 cooperate together with Slug to exert transcriptional activation via H3K9me2 demethylation and H3R2me2s on the vimentin promoter, as well as transcriptional repression through H3K4me2 erasure and H4R3me2s on the E-cadherin promoter. Covalent histone modifications are generally associated with either repression or activation of transcription and play an important role in carcinogenesis [32, 33]. H3R2me2s and H3K4me3 have been shown to simultaneously co-occupy the promoters throughout the mouse genome as markers of active promoters [34]. The Snail-PRMT5-NuRD (MTA1) complex mediates modification of H3R4me2s and deacetylation of histone H3 and induces the transcription repression [23]. However, how PRMT5 and LSD1 drive the epigenetic modification to repress E-cadherin and activate vimentin remains still unknown. It is conceivable that this selectively transcription initiation or repression on target genes by PRMT5 and LSD1 may depend on various molecular partners interacted with them. PRMT5 participates in transcriptional repression by mediating the H4R3me2s when it interacts with several transcriptional corepressors including NuRD, EZH2 or DNMT3A [23, 35, 36]. In contrast, when PRMT5 interacts with Sp1 or OXR1A, it stimulates transcription of target genes by mediating H3R2me2s [29, 37]. Moreover, LSD1 has been reported to exert transcriptional inhibition to coordinate with various epigenetic regulatory complexes such as CoREST, NuRD, CtBP and SIN3A/HDAC via its demethylase activities to H3K4me2 [16, 38, 39]. On the contrary, LSD1 interacts with the ERRα or AR to stimulate the transcription activation through its demethylase activities toward H3K9me2 [30, 40]. Thus, we do not exclude the possibility that other factors may involve in the Slug, PRMT5 and LSD1 mediated transcriptional activation and inhibition for E-cadherin and vimentin, which are still unclear and deserves further study.

Quite a few transcription regulators work in similar fashion to modulate gene expression. Another EMT inducer Twist1 has been found to interact with SET8 to participate in transcriptional repression and activation on the promoters of the Twist1 target genes E-cadherin and N-cadherin via catalyzing H4K20 monomethylation [41]. Earlier studies showed that zinc finger transcription factor ZEB1 uses different co-repressor complexes including CtBP, NuRD or BRG1 to achieve transcriptional repressive activity to target genes during EMT process [42–44]. ZEB1 also forms a complex with a transcriptional co-activator YAP1 to trigger the expression of EMT genes [45, 46]. Snail1 interacts with a variety of co-repressor complexes such as NuRD, LSD1/CoREST, and Sin3A-HDAC1/2 to exert its repressor function during the EMT process [16, 23, 47]. Snail also activates the expression of several key EMT genes including FN1, ZEB1 and MMP9 [48, 49]. It is interesting that these EMT transcription factors seem to employ the dual mode of transcriptional regulation, like slug, a feature that might not be accidental as it offers unique advantages in facilitating the switch of EMT markers. Understanding of the underlying mechanism will provide profound insights into the epigenetic regulation of EMT and metastasis.

Tumor metastasis is the major cause of death in cancer patients. Metastatic breast cancer is generally considered intractable. EMT has been well studied to be a vital trigger of cancer metastasis and correlated with the generation of cancer stem cells and drug resistance, which makes it an attractive therapeutic target for metastatic cancer patients [50]. EMT transcription factors containing Snail, Slug, Twist1/2 and ZEB1/2 are key drivers of the EMT process. And the elevated expression of these proteins contributes to tumor metastasis and is associated with worse prognosis of cancer patients [1]. However, the development of drugs that directly target transcription factors remains challenging currently. Our study suggests that PRMT5 and LSD1 function as a dual epigenetic modifier to promote Slug induced EMT program, suggesting an opportunity for treatment of metastatic breast cancer by co-targeting of PRMT5 and LSD1. Our study revealed that PRMT5 and LSD1 are synchronously expressed in breast cancer patients and synergistic inhibition of PRMT5 and LSD1 significantly slows down the breast tumor growth and metastasis, providing a novel promising therapeutic strategy for patients with metastatic breast cancer.

## Conclusion

In this study, we found that Slug works with PRMT5 and LSD1 to act as a dual epigenetic regulator on the promoters of E-cadherin and vimentin. We demonstrated that PRMT5 and LSD1 cooperate to promote the EMT and invasion of cancer cells, and that PRMT5 expression is positively correlated with LSD1 expression in breast carcinoma specimens. Our data suggest that the combination of LSD1 inhibitor (SP2509) and PRMT5 inhibitor (EPZ015666) synergistically lead to a strong reduction of vimentin expression, induction of E-cadherin expression and decreased invasion of breast cancer cells in vitro, and also efficiently inhibit tumor growth and lung metastases of breast cancer in vivo. Our findings provide a promising therapeutic strategy for the treatment of patients with metastatic breast cancer.

## Supplementary Information

**Additional File 1.** 

## Data Availability

All data obtained and/or analyzed in this study were available from the corresponding author in a reasonable request.

## Abbreviations

EMT: Epithelial to Mesenchymal Transition
PRMT5: Protein Arginine Methyltransferase 5
LSD1: Lysine-specific histone demethylase 1
IHC: Immunohistochemistry
TCGA: The Cancer Genome Atlas
3’UTR: 3'-Untranslated region
5’UTR: 5'-Untranslated region
ChIP: Chromatin Immunoprecipitation
Re-ChIP: Re-chromatin immunoprecipitation
qPCR: Quantitative real-time polymerase chain reaction
RT-PCR: Reverse transcription- polymerase chain reaction
Go analysis: Gene Ontology analysis
