# Supplementary material for: The PRMT5-LSD1 axis confers Slug dual transcriptional activities and promotes breast cancer progression
Source: J Exp Clin Cancer Res. 2022 Jun 2;41:191. doi: 10.1186/s13046-022-02400-7 (PMC9164399; doi:10.1186/s13046-022-02400-7)
Supplement: Supplementary file 1 — Additional File 1. [file 13046_2022_2400_MOESM1_ESM.pdf]

**The PRMT5-LSD1 axis confers Slug dual transcriptional activities and promotes breast cancer progression**

Jianchao Zhang[1*], Xiaokai Fan[2], Yunfan Zhou[1], Liang Chen[2*] and Hai Rao[1*]

1, Department of Biochemistry, School of Medicine, Southern University of Science and Technology, Shenzhen, China

2, Shenzhen Laboratory of Tumor Cell Biology, Center for Protein and Cell-based Drugs, Institute of Biomedicine and Biotechnology, Shenzhen Institute of Advanced Technology, Chinese Academy of Sciences, Shenzhen, China

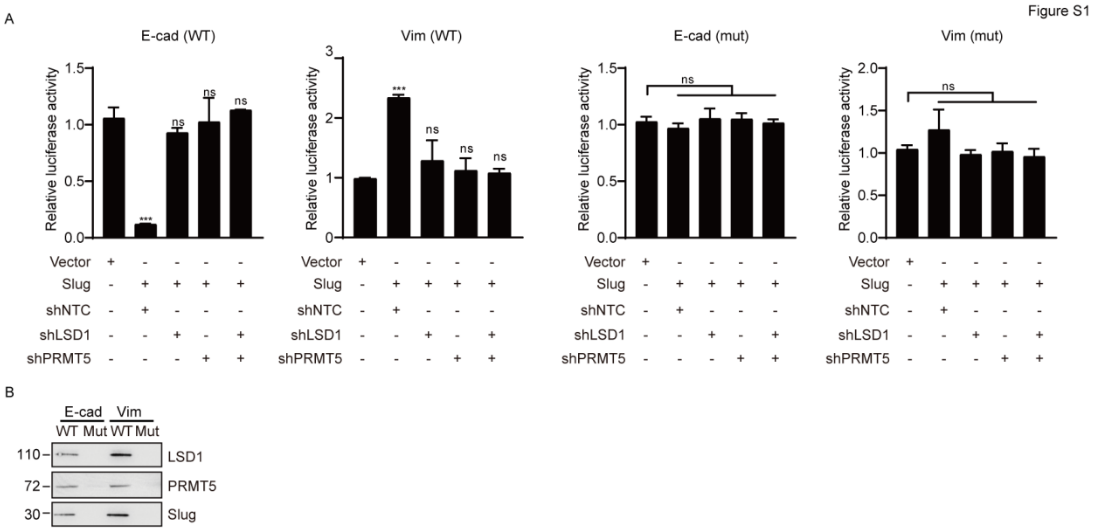


**Figure S1. Characterization of the Slug-PRMT5-LSD1 complex binding motif in the E-cadherin and vimentin promoter.**

(A) E-cadherin or vimentin promoter luciferase construct (WT) as well as their mutants (mut) were coexpressed with indicated plasmids in HEK293T cells. After 48h, luciferase activity was measured. Relative luciferase activity was calculated as firefly luciferase activity divided by renilla luciferase activity and shown relative to the control. Data are Means ± S.D. from triplicate analyses. ***P< 0.001, as determined by Student’s t test (B) The Slug-PRMT5-LSD1 complex purified from HEK293T cells expressing Flag-Slug, HA-PRMT5 and HA-LSD1 were incubated with biotinylated double-stranded wild-type (CCCAAA) or mutated (TCTGAG) oligonucleotides followed by pull-down and immunoblotting with antibodies against Flag or HA.

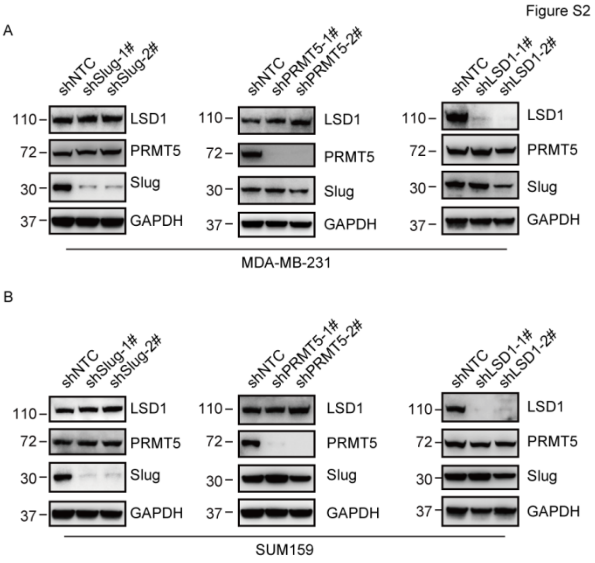


**Figure S2. Evaluation the knockdown effects of each shRNA in breast cancer cells.**

Expression of PRMT5, LSD1 and Slug was analyzed by Western blotting in MDA-MB231 (A) or SUM159 (B) cells with stable shNTC or shRNAs against Slug, PRMT5, and LSD1.

Figure S3

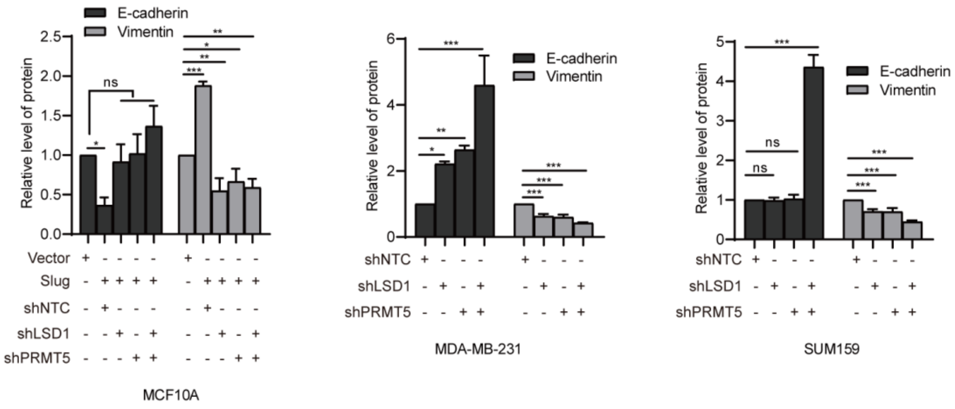


**Figure S3. Quantification of E-cadherin and vimentin from western blot in Figure 4D (left), 4G (middle) and 4I (right) (n=3; error bars indicate SD, *p<0.05,**p<0.01, ***p< 0.001, Student's t-test).**

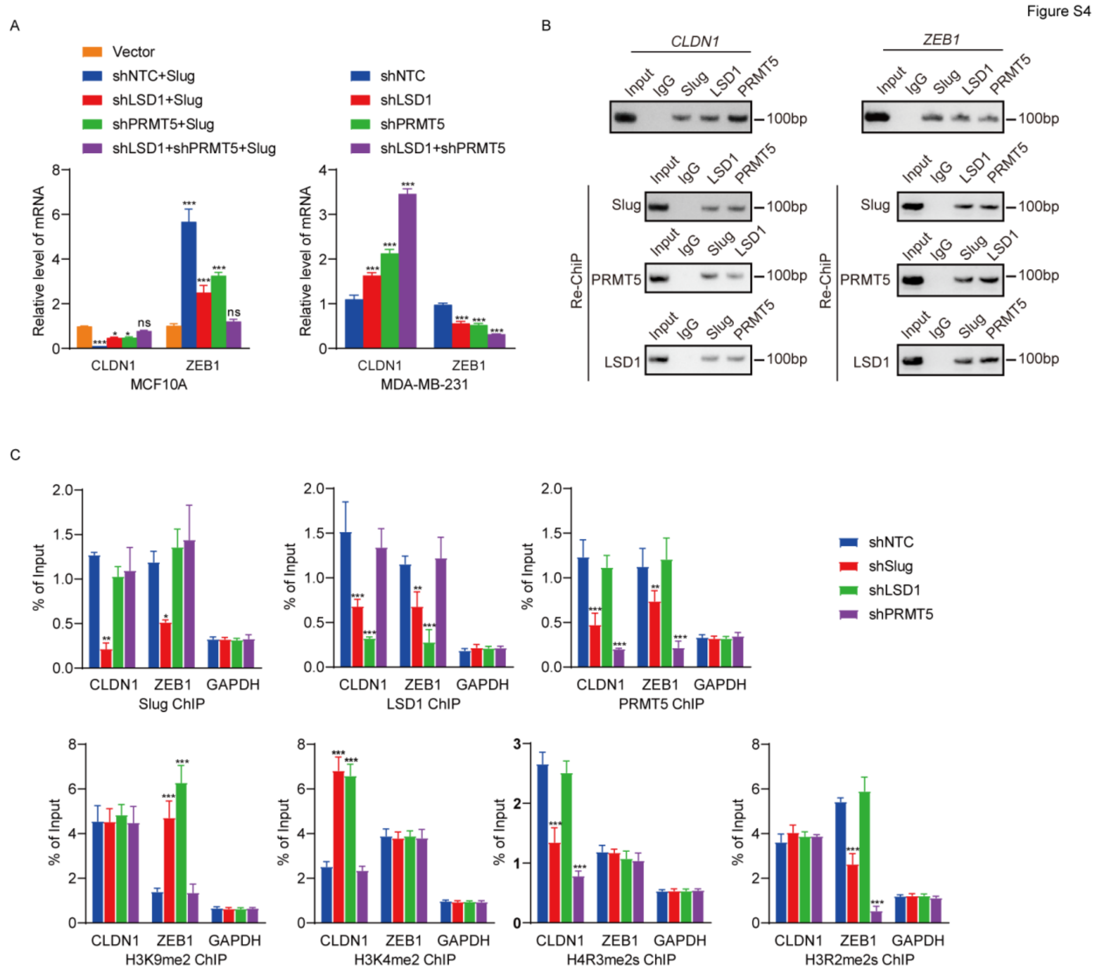


**Figure S4. Slug cooperates with PRMT5 and LSD1 to regulate the transcription of Claudin-1 (CLDN1) and ZEB1.**

(A) MCF10A cells were infected with lentiviruses carrying the indicated shRNAs together with Slug expression constructs or empty vector. The mRNA level of indicated genes was measured by qPCR (left). MDA-MB-231 cells were infected with lentiviruses carrying shNTC, shLSD1, shPRMT5 and shLSD1+shPRMT5. The mRNA level of indicated genes was determined by qPCR (right). (B) ChIP and Re-ChIP experiments were done in MDA-MB-231 cells with the indicated antibodies. (C) MDA-MB-231 cells were infected with lentiviruses carrying the indicated shRNAs, and the association of Slug, PRMT5, LSD1, H4R3me2s, H3R2me2s, H3K9me2 and H3K4me2 at the promoter of CLDN1 and ZEB1 was analyzed with the qChIP assay. For A and C, the data represent the mean ± SD from three separate experiments (*p<0.05, **p<0.01, ***p< 0.001, Student's t-test).

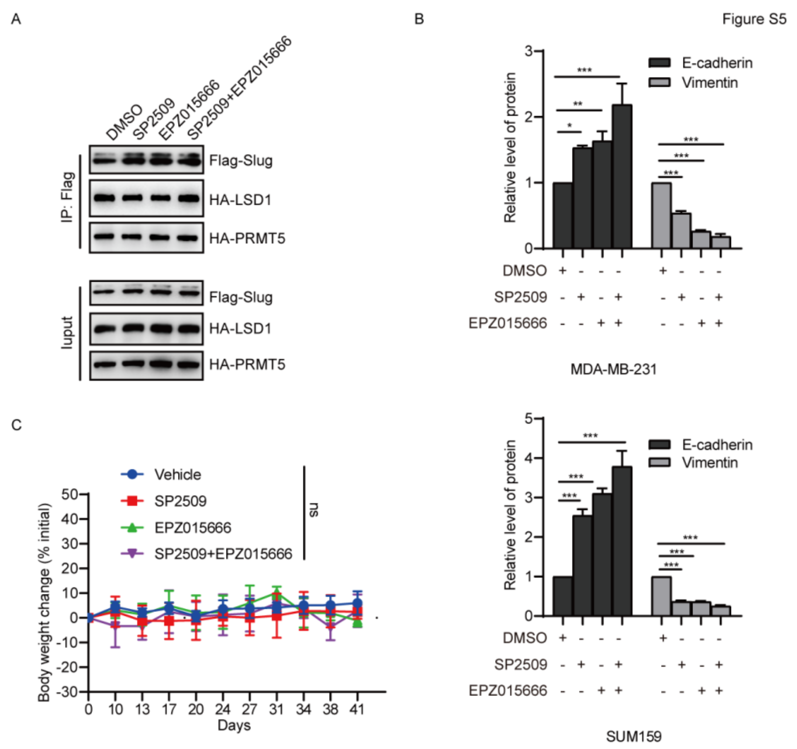


**Figure S5. The SP2509 or EPZ015666 had no effect on body weight of mice and interaction of Slug-PRMT5-LSD1 complex.**

(A) Flag-Slug, HA-PRMT5 and HA-LSD1 were co-expressed in HEK293T cells, respectively. After 24h, the cells were treated with DMSO, SP2509 (1 μM), EPZ015666 (1 μM) or SP2509 plus EPZ015666 for 72h. Cell lysates were immunoprecipitated with Flag antibody followed by immunoblotting with the indicated antibodies. (B) Quantification of E-cadherin and vimentin from western blot in Figure 5A (upper) and 5B (lower) (n=3; error bars indicate SD, *p<0.05, **p<0.01, ***p< 0.001, Student's t-test.). (C) The body weight change of the mice treated with Vehicle, SP2509 (25 mg/kg, i.p.), EPZ015666 (100 mg/kg, i.p.) and the combination of SP2509 and EZP015666 (n=5) starting from day 10 post transplantation. The drugs were administered on days 10, 13, and 17 in an eight days repeating cycle for three cycles.

A

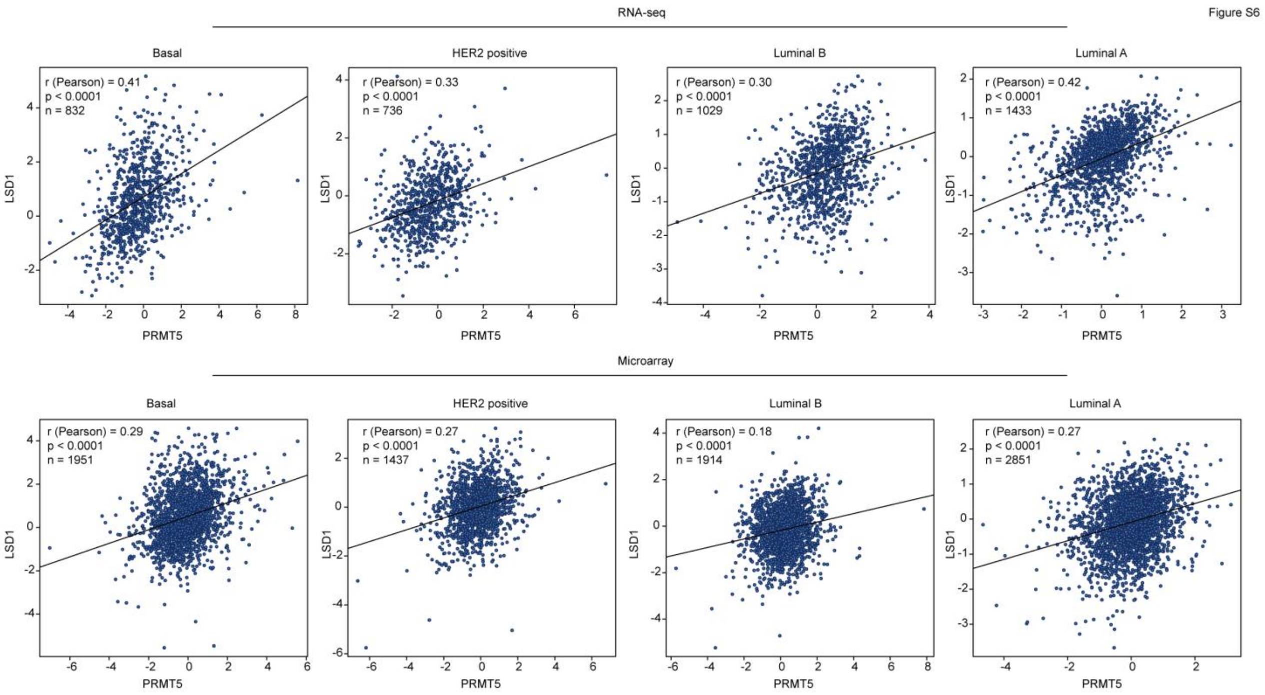


B

Co-expression of PRMT5 and LSD1 in different subtypes of breast cancer specimens

| Tumor type | LSD1 | PRMT5 Low | PRMT5 Middle | PRMT5 High | Total | r(Pearson) | P value |
|---|---|---|---|---|---|---|---|
| Luminal | Low | 9 | 8 | 4 | 21 | 0.5792 | <0.001 |
| | Middle | 3 | 11 | 4 | 18 | | |
| | High | 1 | 4 | 26 | 31 | | |
| | Total | 13 | 23 | 34 | 70 | | |
| Her2+ | Low | 5 | 1 | 1 | 7 | 0.5040 | 0.033 |
| | Middle | 0 | 1 | 3 | 4 | | |
| | High | 1 | 2 | 4 | 7 | | |
| | Total | 6 | 4 | 8 | 18 | | |
| Basal | Low | 4 | 4 | 0 | 8 | 0.6242 | <0.001 |
| | Middle | 2 | 3 | 0 | 5 | | |
| | High | 1 | 4 | 8 | 13 | | |
| | Total | 7 | 11 | 8 | 26 | | |

**Figure S6. The correlation between PRMT5 and LSD1 in different subtypes of breast cancer specimens.**

(A) Analysis of public clinical datasets for the expression of PRMT5 and LSD1 in an RNA-sequencing dataset (upper) and a DNA microarray dataset (lower) with indicated breast cancer subtype. The relative mRNA level of LSD1 was plotted against that of PRMT5. Correlations were analyzed using Pearson correlation method. (B) IHC analysis of the relationship between LSD1 and PRMT5 expression in indicated breast cancer subtype. The association was assessed by Chi-square analysis, and the strength of correlation was evaluated by Pearson correlation coefficient. Statistical significance was defined as $p<0.05$.
